# Changes in attentional breadth scale with the demands of Kanizsa-figure object completion–evidence from pupillometry

**DOI:** 10.3758/s13414-023-02750-0

**Published:** 2023-07-05

**Authors:** Leonie Nowack, Hermann J. Müller, Markus Conci

**Affiliations:** Department of Psychology, Ludwig-Maximilians-Universität München, Leopoldstr. 13, D-80802 München, Germany

**Keywords:** Perceptual grouping, Object integration, Visual attention, Pupillometry, Covert attention, Attentional breath

## Abstract

The present study investigated whether the integration of separate parts into a whole-object representation varies with the amount of available attentional resources. To this end, two experiments were performed, which required observers to maintain central fixation while searching in peripheral vision for a target among various distractor configurations. The target could either be a “grouped” whole-object Kanizsa figure, or an “ungrouped” configuration of identical figural parts, but which do not support object completion processes to the same extent. In the experiments, accuracies and changes in pupil size were assessed, with the latter reflecting a marker of the covert allocation of attention in the periphery. Experiment 1 revealed a performance benefit for grouped (relative to ungrouped) targets, which increased with decreasing distance from fixation. By contrast, search for ungrouped targets was comparably poor in accuracy without revealing any eccentricity-dependent variation. Moreover, measures of pupillary dilation mirrored this eccentricity-dependent advantage in localizing grouped targets. Next, in Experiment 2, an additional attention-demanding foveal task was introduced in order to further reduce the availability of attentional resources for the peripheral detection task. This additional task hampered performance overall, alongside with corresponding pupil size changes. However, there was still a substantial benefit for grouped over ungrouped targets in both the behavioral and the pupillometric data. This shows that perceptual grouping scales with the allocation of attention even when only residual attentional resources are available to trigger the representation of a complete (target) object, thus illustrating that object completion operates in the “near absence” of attention.

## Introduction

**Fig. 1:**
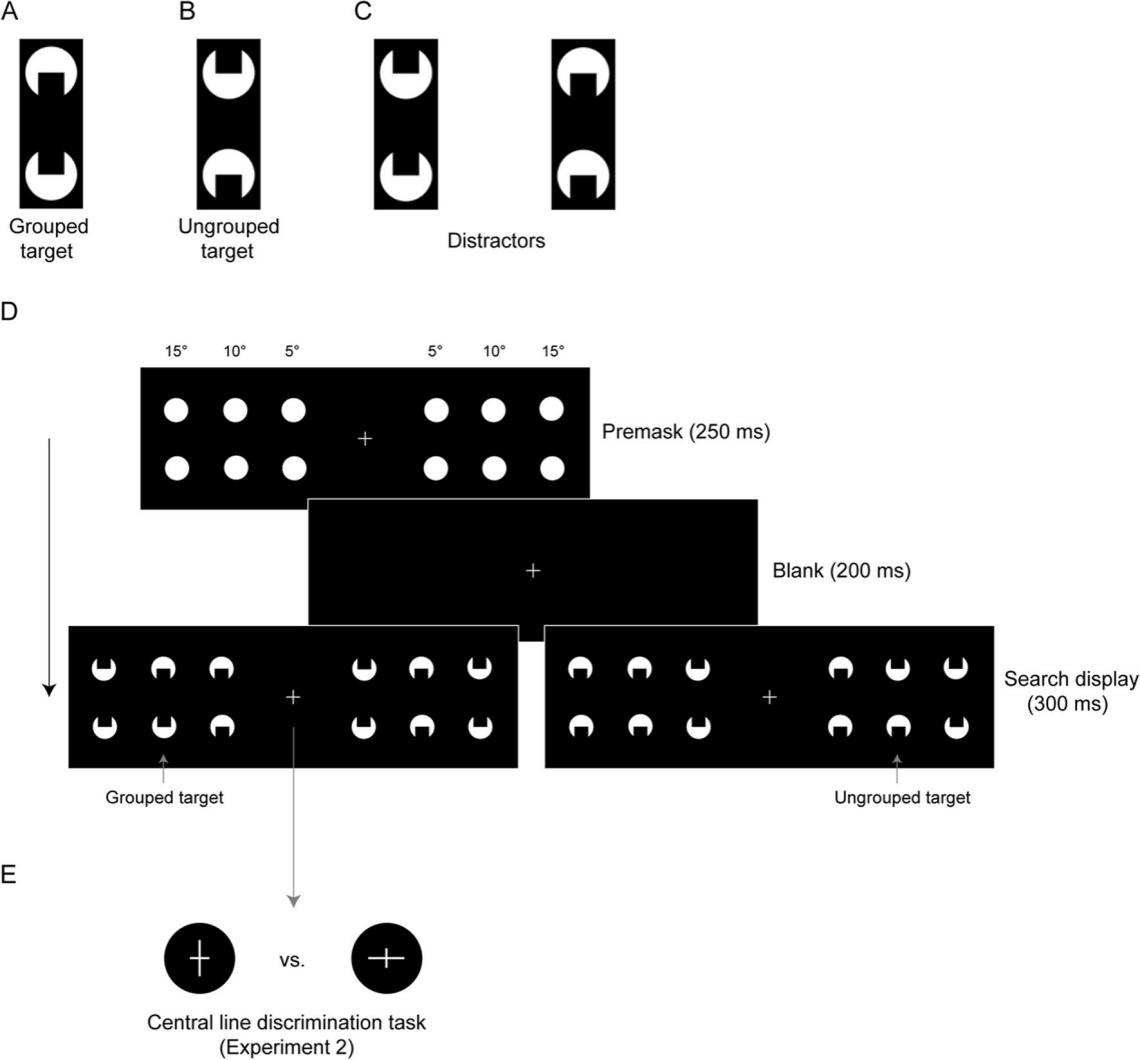
Illustrations of the grouped (**A**) and ungrouped (**B**) targets and the distractor configurations (**C**), as presented in the experiments. Panel (**D**) depicts an example trial sequence in Experiment 1. A premask display presented six filled placeholder circles for 250 ms, which was followed by a blank screen for 200 ms. Next, the search display appeared and remained on the screen for 300 ms, either presenting a grouped (left) or an ungrouped (right) target (in the example depicted, both targets are presented at an eccentricity of 10º). (**E**) In Experiment 2, the trial sequence was the same, except that an additional, foveal task was added to the search display, which required a line length discrimination of the (vertically or horizontally stretched) fixation cross

The visual system has developed dedicated mechanisms that structure and organize the complex visual input that we are constantly exposed to in everyday life. One such mechanism, serving the integration of fragmented image parts into coherent, whole “objects,” is perceptual grouping. By implementing a set of organizational principles, grouping processes structure the perceptual input, combining fragments into coherent wholes and segmenting objects from each other as well as the background (Koffka, 1935; Wertheimer, 1923). One example that illustrates these mechanisms of object integration is the so-called *Kanizsa figure* (Kanizsa, 1976); see Fig. 1A for an example. In this configuration, the arrangement of the circular “^pacman^” inducer elements creates the vivid impression of an “illusory” rectangle that lacks a corresponding physical object.

Object integration by means of perceptual grouping appears to be achieved in a fairly effortless manner. However, whether object completion operates automatically or whether it depends on the engagement of attention is a matter of intense debate. Influential accounts such as Feature Integration Theory (Treisman & Gelade, 1980) assume that attention must first be allocated to a given stimulus in order to enable part-to-whole integration and render complete-object representations. In this view, perceptual grouping would generate a coherent whole object only when focal attention is allocated to the object’s location. Opposing theories posit that the representation of complete objects arises “preattentively,” prior to the engagement of attention (Driver & Baylis, 1998; Humphreys et al., 1994; Scholl, 2001). Major support for the latter, “object-based” view of attention comes from studies that tested object-completion mechanisms in neuropsychological patients with parietal brain damage and associated deficits of selective attention in one side of the visual field. While these patients would typically miss targets in the impaired visual field, access to such “neglected” targets can be substantially improved by providing a grouped structure that links the attended with the unattended region across the two visual hemifields (e.g., Conci et al., 2009; Mattingley et al., 1997). Importantly, however, our recent studies show that such a benefit for grouped objects depends crucially on the availability of attention: these patients exhibited enhanced target detection in the impaired hemifield only when attention was available to spread into the impaired visual field, but not when it was engaged in the unimpaired visual field (Conci et al., 2018; Gögler et al., 2016; Nowack et al., 2021). This in turn supports the view that object completion requires the availability of at least some residual amount of attentional resources.

Following these findings from neuropsychological patient studies, the present experiments investigated whether part-to-whole object integration and search guidance by salient, integrated objects would likewise scale with the amount of available attentional resources in healthy participants. Methodologically, our study made use of pupillometry (the measurement of pupil diameter), since variations of pupil size have been shown to also reflect (higher-level) information processing (see, e.g., Eberhardt et al., 2021), including the allocation of visuo-spatial attention. The latter is evidenced by findings of a close relationship between the pupillary light reflex and concurrent attention shifts (for reviews, see Laeng & Alnaes, 2019; Mathôt, 2018). For instance, covert shifts of attention towards a bright (or, respectively, dark) stimulus in the periphery consistently evoke a pupillary constriction (or, respectively, dilation), demonstrating that changes in pupil size can be used to track where attention is allocated (Binda et al., 2013; Mathôt et al., 2013, 2014; Naber et al., 2013).

In fact, pupil-size measures may also serve as markers of the allocation of covert attention to peripheral stimuli in the absence of any luminance manipulations, as shown by Brocher et al. (2018). In their study, two peripheral stimulus configurations, consisting of four objects each, were presented bilaterally at varying distances from (central) eye fixation (though with both configurations being equidistant from fixation). Observers fixated the screen center and identified the lateral configurations via covert shifts of attention. After the onset of the stimuli, observers were presented with a central arrow cue that pointed towards one side of the display, and their task was to report the number of targets (white triangles) on the cued side. The results revealed performance accuracy to decrease with increasing eccentricity (ranging from 12.5º up to 42.5º). Importantly, the increase in task difficulty with eccentricity was associated with stronger pupil dilations for more peripheral stimuli, suggesting that pupil size not only reflects task difficulty (e.g. Beatty, 1982), but also covert shifts of attention to the target(s) without a concurrent change in luminance (see also Hüttermann & Memmert, 2017; Hüttermann et al., 2013, 2014). In a more recent experiment, Ivanov et al. (2019) also measured changes in pupil size in response to attention shifts. Participants were presented with tilted Gabor patches, three on the left and three on the right side of fixation (at varying eccentricities). Following a bilateral peripheral location cue, observers were asked to indicate the orientation of the two cued “target” Gabor patches (one on each side of the display, with both targets being equidistant from fixation and depicting the same orientation). The results again showed that pupil size increased with increasing eccentricity of the attended locations. These findings consistently show that pupil size is linked to attentional shifts or, respectively, the “breadth” of attention: the pupil becomes wider when attention is allocated to more peripheral locations, that is, when attention is distributed more broadly across the visual field as compared to when a more central focus is required (Brocher et al., 2018; Daniels et al., 2012; Ivanov et al., 2019; Klatt et al., 2021; Mathôt & Ivanov, 2019; see Mathôt, 2020, for a review).

Of note, in many instances, the detection of a target in the periphery is not only associated with a broader or narrower focus of attention (e.g., depending on stimulus eccentricity), but more peripheral stimuli usually also result in a concurrent increase in task effort (e.g., as also shown in Brocher et al., 2018). Moreover, task effort has also been associated with an increase in pupil size (for a review, see, e.g., Beatty, 1982), which makes it difficult to disentangle the degree to which changes in pupil size are related to task effort and/or attentional breadth. While both effort and attention are indeed intricately linked, they appear to describe performance at different levels of processing: For example, search for a salient, grouped target is typically less effortful than search for a non-salient, ungrouped target (Conci et al., 2007), and as such, this difference in performance would reflect variations of task effort. However, when assuming that these variations in performance elicit concurrent variations of attention, then the observed pattern of results would concurrently reflect changes in the distribution of attention (as a function of task difficulty). Essentially, this distinction is comparable to classical assumptions in visual search, where the slopes of reaction times across set sizes are associated with search efficiency (Treisman & Gelade, 1980). That is, steeper search slopes denote more inefficient (that is, more effortful) search performance, and more inefficient search is in turn associated with a narrower attentional focus (that requires the serial scanning of the display). Conversely, flat search slopes would typically be associated with more efficient search and a broader tuning of the attentional spotlight. Task difficulty and/or mental effort thus appear to be directly related to attentional breadth, with both processes also being reflected in the (objective) measurement of pupil size.

In the current study, we adopted this widely accepted logic and applied it to variations of pupil size (which constitutes a more objective measurement of performance than slopes of reaction time functions). Given this, pupil-dilation measures were used as a marker for (i) task effort and (ii) the concurrent allocation of visual attention to peripheral stimuli that vary in their demands for object integration. In our experiments, a visual search task presented variants of Kanizsa figures as target and distractor configurations, that were roughly comparable to those used in previous studies (Conci et al., 2007; Conci et al., 2011; Nie et al., 2016; Nowack et al., 2021; Wiegand et al., 2015). Importantly, the target could vary in terms of its grouping strength: it could be either a complete object, namely, an illusory Kanizsa-type rectangle (grouped target, Fig. 1A), or a physically identical, symmetrical configuration but without inducing an illusory figure (ungrouped target, Fig. 1B). The distractors presented together with the target in the display were non-symmetric arrangements that were equally similar to both types of target (Fig. 1C). A given display (Fig. 1D) consisted of six candidate target configurations – three to the left and three to the right of the central fixation cross at varying eccentricities (5°, 10°, and 15°). In Experiment 1, participants were required to maintain central fixation and localize the lateral target item, which was positioned randomly at any of the three possible eccentricities in one or the other display half, thus putatively requiring attention to either focus more centrally or to broaden the focus more towards the periphery in order to report the (left/right) hemifield in which the target appeared. In Experiment 2, targets were only displayed at the intermediate (i.e., 10°) position while attention was additionally engaged, at least to some degree, in a second, foveal line-discrimination task (Fig. 1E), comparable to the procedure used in previous studies (e.g., Mack et al., 1992; see also Li et al., 2002; Moore & Egeth, 1997).^1^ The adoption of a foveal attention-demanding task allowed us to further test whether the detection of a grouped versus an ungrouped target depends on the amount of attentional resources that are currently available.

Previous search studies with Kanizsa figures showed search efficiency (i.e. task effort) to be higher (Conci et al., 2007; Nie et al., 2016) and attention allocation to be faster for grouped as compared to ungrouped target configurations (Chen et al., 2019; Conci et al., 2011; Wiegand et al., 2015), consistent with attentional guidance improving with an increase of the grouping strength in the target. However, it is not clear whether the allocation of covert attention to a given target at varying distances from fixation, as reflected in pupil-dilation measures, would scale with such target-related grouping demands. Moreover, if attention is engaged to a large extent in a second, foveal task, its allocation to the peripheral target should be hampered by this limitation of attentional resources – which should again be reflected in pupil-dilation measures.

## Experiment 1

Experiment 1 used a visual search task, which was optimized to measure variations in pupil dilation (for a methodological overview, see Mathôt & Vilotijević, 2022). The experiment was performed to examine whether a narrow or broadly distributed focus of attention influences object integration for grouped versus ungrouped target items at varying eccentricities (of 5º, 10º, and 15º). As depicted in Fig. 1D, observers were presented with a linear (horizontal) array of six stimulus configurations, three to the left and three to the right of central fixation; their task was to indicate whether one of two possible target configurations appeared on the left or the right side (among the five distractor configurations). Observers were instructed to maintain central fixation throughout a given trial (checked by an eye tracker). Accordingly, correctly (left/right) localizing the target was assumed to require a narrower or more broadly tuned attentional focus. Both performance-accuracy and pupil-dilation measures (the latter serving as a marker for variations of the attentional breadth; see, e.g., Ivanov et al., 2019) were obtained to determine how object completion affects the processing of the target item in the periphery.

## Materials and method

### Participants

Thirty participants (ten males; mean age 28.03 (*SD =* 7.22) years) with normal or corrected-to-normal vision took part in the experiment. One participant, however, had to be excluded from the Pupillometry analysis due to problems with the eye-tracker recording. Participants (mainly Psychology students) received either monetary compensation (9 €) or course credits for taking part in the experiment. The experimental procedure was approved by the local ethics committee (Department of Psychology, Ludwig-Maximilians-University Munich), and written informed consent according to the Declaration of Helsinki was obtained from all participants prior to the experiment.

Sample size was determined on the basis of an a priori power analysis, which aimed for 95% power to detect a minimum *f*(*U*) effect size of 0.35 (partial η^2^ = .11) at an alpha level of .05 and a nonsphericity correction of 1. This effect size was determined on the basis of previous studies that used a comparable task and similar stimulus configurations (Conci et al., 2018; Nowack et al., 2021). An influence of attention on object integration (in a within-subjects design) would be reflected by a significant two-way Target Configuration by Eccentricity interaction, which, according to our power analysis, would require N = 16 participants. However, pupil size effects are typically rather small and previous pupillometry studies therefore typically used larger sample sizes (see, e.g., Brocher et al., 2018; Ivanov et al., 2019). Given this, we decided to (almost) double the sample size and to test a total of N = 30 participants.

### Apparatus

The experiment was programmed with the Psychophysics and Eyelink toolboxes (Kleiner et al., 2007) running in Matlab (MATLAB, 2017). Participants viewed the display screen (19-in. monitor, 1,024 × 768 pixels resolution, 85-Hz refresh rate) from a distance of approximately 57 cm and their viewing position was stabilized by means of a forehead-and-chin rest. Eye movements were recorded (at a sampling rate of 1,000 Hz) from the right eye using an Eyelink CL eye-tracker system (SR-Research Ltd., ON, Canada). At the beginning of each block, a five-dot calibration routine was performed. Eye-movement monitoring was intended to ensure that participants’ gaze remained fixated at the screen center throughout the entire trial (i.e., until a manual response was provided). A given trial was discarded if participants moved their gaze more than 2° away from the central fixation cross, which occurred in 1.9% of all trials.

### Stimuli

**Fig. 2:**
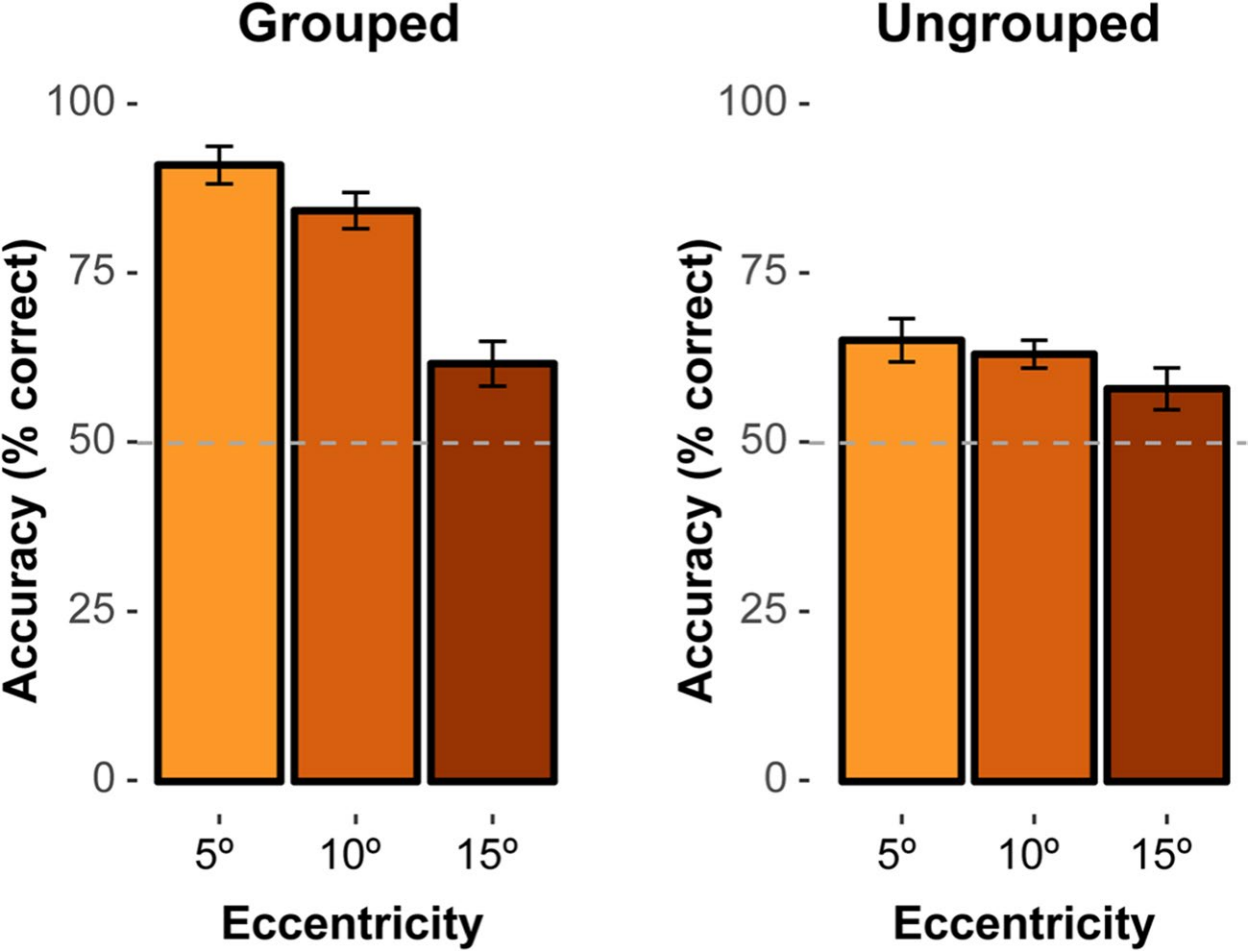
Mean accuracies (% correct), with within-subject 95% confidence intervals, for grouped (**left**) and ungrouped (**right**) targets as a function of target eccentricity

All six stimulus configurations consisted of two white circles (luminance: 1.83 cd/m^2^) with a radius of 1° of visual angle, which were presented on a black background (luminance: 0.01 cd/m^2^). Each two-circle configuration was arranged vertically, subtending 1° × 2.6° of visual angle. In each circle, a square-shaped indent (0.4° × 0.4°) was removed from the top or the bottom, thus forming a “pacman” inducer element. The grouped target (Fig. 2A) was an arrangement with both indents facing towards the “inside” (i.e., the horizontal midline of the screen), which generated a vivid impression of a symmetrically organized, illusory Kanizsa rectangle. For the ungrouped target (Fig. 2B), both indents were arranged to face “outwards” (i.e., away from the midline), which also resulted in a symmetrical configuration, but without the emergence of an illusory object. Finally, distractor configurations (Fig. 2C) consisted of a pair of circles with both indents removed from either the top or the bottom, so that no illusory figure could be formed. Stimuli were presented at six lateral positions, three to left and three to the right of the central white fixation cross at eccentricities of 5º, 10º, and 15º, respectively (fixation cross: size 0.4º × 0.4º). Within a given trial display, either a grouped or an ungrouped target would be presented with equal probability at one of the six possible locations; the remaining five locations were occupied by a distractor configuration, with an upward or downward orientation of both inducers (orientations were randomly selected for each distractor position). Prior to the search display, a premask display presented complete white circles at the same locations as the subsequent pacman inducer elements (see Fig. 2D for an example trial sequence).

### Procedure

The experiment was conducted in a dimly lit, soundproof experimental room. Participants were instructed to fixate the cross in the screen center for the entire trial duration and localize the target in the left/right half of the display (which was assumed to require changes of the attentional breadth for stimuli at the peripheral locations), responding to any target detected at any of the three positions in the left/right visual field with the left/right arrow key on the keyboard. Participants were asked to respond as accurately as possible, without any time restriction.

The experiment consisted of four blocks in total with short breaks in between. Each block presented 120 trials. Two consecutive blocks presented a grouped target and the other two blocks an ungrouped target. The order of presentation of the grouped/ungrouped target blocks was counterbalanced across participants. Grouped and ungrouped targets were presented in a blocked fashion to ensure that observers could prepare specifically for a given target stimulus. The position of the target (at the various eccentricities in the left or right display half) was randomized across trials so that participants could not direct attention to the target location beforehand, thus requiring a rather broad attentional focus at the beginning of each trial. Each block presented 20 trials for each position and display half, yielding 480 trials in total.

Each trial started with the presentation of the fixation cross for 250 ms. Next, the premask was presented for 250 ms, followed by a blank screen shown for 200 ms. Subsequently, the search display was presented for 300 ms. After the offset of the search display, a “blank” screen with only the fixation cross remained in view for 1,750 ms, providing sufficient time for the pupil dilation to be measured (e.g., Brocher et al., 2018). Following the dilation period, a response display was presented, which depicted the word “Where was the target?” at the center of the screen and which was presented until participants provided their manual response by pressing the left/right arrow key. Altogether, a given trial lasted on average 3,724 ms (mean response time of 3,124 ms plus 700 ms before search display onset), thus providing sufficient time for the pupillary response to come back to normal before the next search display would be presented. An example trial sequence is depicted in Fig. 1D. The experiment lasted approximately 1 h in total, including the instruction of the participants, a short practice session, and the eye-tracker calibration routine at the beginning of each block. Observers were asked to complete at least ten trials per target type. However, the experimenter also informed them and ensured that observers would practice the task until they were familiar with the experimental procedure. The actual experiment then started when observers felt comfortable with the task.

### Pupillometry

The raw eye-tracking data from all participants was exported into a text-format sample report using the EyeLink DataViewer (EyeLink Data Viewer, 2007). For all preprocessing steps as well as the statistical analysis of the pupil-size (and response-accuracy) data, we used R Studio (RStudio Team, 2015). For the analyses of the pupillary responses, trials with incorrect behavioral responses were discarded from the data proper. We also excluded trials on which the pupil-size measure was larger than three standard deviations from the overall mean, trials which yielded fewer than 60% of useable data points because of blinks (note that this criterion resulted in the exclusion of only 0.4% of all trials), and trials in which overt eye movements were made (see Brocher et al., 2018; Mathôt et al., 2018). In total, 4.6% of all trials were excluded using this elimination procedure (6.1% in Experiment 2). Note that the eye tracker failed to record data for one participant (Experiment 1), and, hence, the pupillometry analyses presented below are based on a sample of 29 observers.

Pupil size was calculated by means of a subtractive baseline correction (for a similar procedure, see Brocher et al., 2018; Mathôt et al., 2018). Thus, for each trial and participant, we extracted the maximum pupil size during the 250-ms interval when the premask display was presented (baseline), and then subtracted this baseline measure from the maximum pupil size after the presentation of the stimulus display during the 1,750 ms dilation period (i.e., after search-display offset).

## Results

### Response accuracy

Trials on which participants did not maintain central fixation were excluded from the analysis (1.9% of all trials). Overall, the mean percentage of correct responses was 70.5%. Figure 2 presents the mean accuracies as a function of eccentricity, separately for the two types of target configuration. Individual mean accuracies were analyzed using a repeated-measures analysis of variance (ANOVA) with the factors Target Configuration (ungrouped, grouped) and Eccentricity (5º, 10º, 15º). Greenhouse-Geisser corrected values are reported in case Mauchley’s test of sphericity was significant (*p* < .05). This analysis revealed a main effect of Target Configuration, *F*(1, 29) = 62.27, *p* < .001, η^2^_G_ = .31, with higher response accuracy for grouped (79.2%) versus ungrouped targets (62.2%). There was also a significant main effect of Eccentricity, *F*(2, 58) = 48.84, *p* < .001, η^2^_G_ = .27: overall, accuracy decreased with increasing distance of the target from fixation (78.2%, 73.8%, 59.9% for eccentricities of 5°, 10°, and 15°, respectively). Importantly, there was also a significant Target Configuration × Eccentricity interaction, *F*(1.54, 44.66) = 24.62, *p* < .001, η^2^_G_ = .12. Holm post hoc tests revealed that for the grouped target, there were significant differences between the 5° (91.1%) and both the 10° (84.4%, *p* = .036) and 15° (61.7%) eccentricities, as well as between the 10° and 15° eccentricities (*p*s < .001). Thus, in the grouped-target condition, accuracy dropped significantly the further away from fixation the target appeared. By contrast, in the ungrouped-target condition, there were no significant differences across the three eccentricities (all *ps* > .05), with the mean response accuracy (62%) being overall comparable to performance in the grouped target condition at the most distant, 15°-eccentricity position, *p* = .791. Together, this pattern shows that an increase in target eccentricity substantially reduced localization accuracy for the grouped target, while the localization of the ungrouped target was less accurate overall (i.e., even at the position closest to fixation) and not modulated further by target eccentricity.

Of note, performance in all conditions was significantly above the 50% chance level, *t*(29)s > 3.58, *p*s < .001. However, in order to further exclude the possibility that the significant interaction was due to the ungrouped targets revealing a floor effect (i.e., with their respective performance levels being somewhat, i.e., some 10%, above chance), we additionally arcsine-transformed the accuracy data to improve normality. The pattern of results for these arcsine-transformed accuracies stayed the same as described above, revealing significant main effects of Target Configuration, *F*(1, 29) = 72.80, *p* < .001, η^2^_G_ = .35, and Eccentricity, *F*(2, 58) = 50.17, *p* < .001, η^2^_G_ = .30, and again a significant Target Configuration × Eccentricity interaction, *F*(2, 58) = 36.40, *p* < .001, η^2^_G_ = .17. Holm post hoc tests also again showed the same pattern as described above. The significant interaction is therefore unlikely to be due to a floor effect in the ungrouped targets.

### Pupillometry

**Fig. 3:**
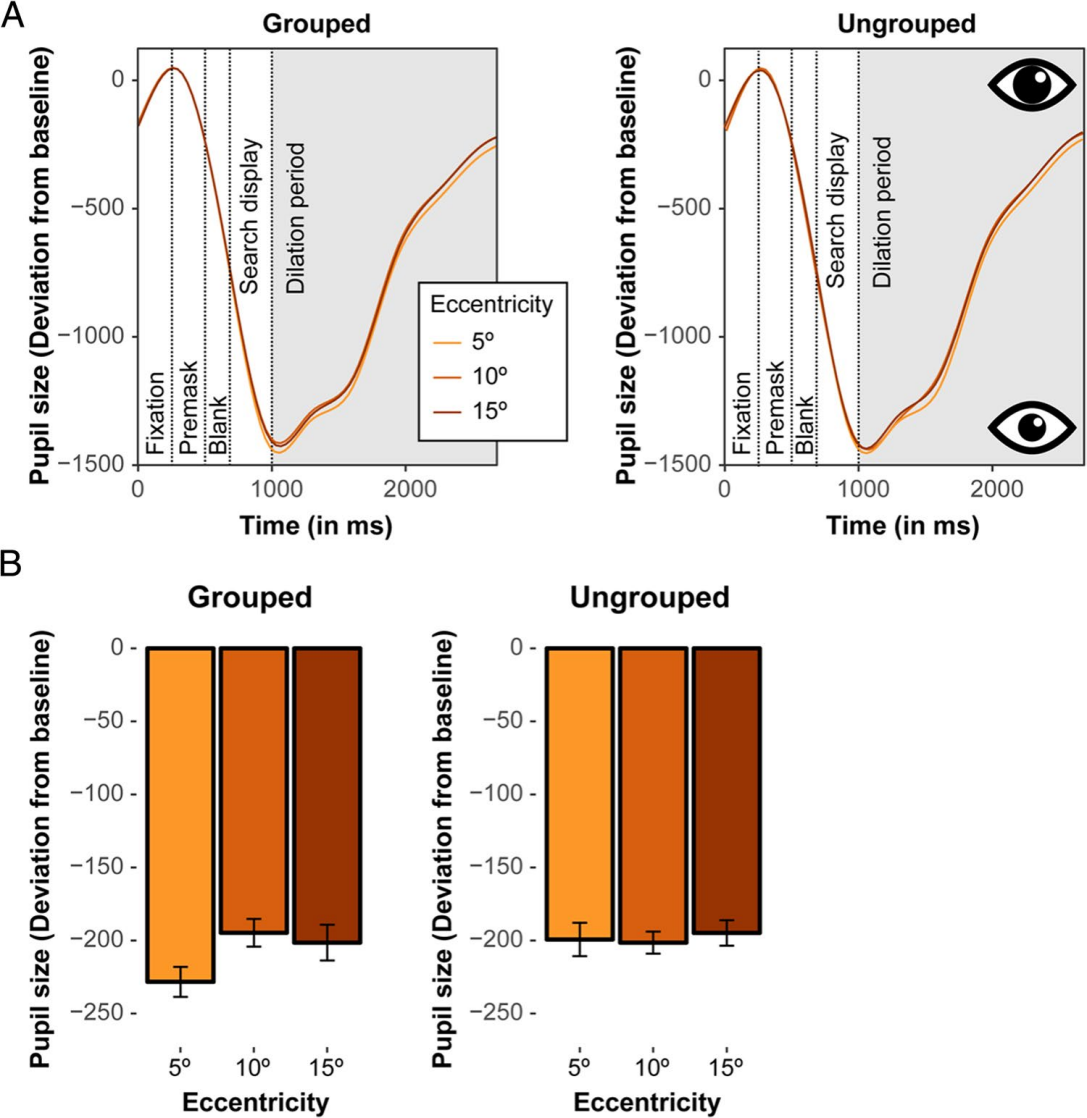
(**A**) Time courses of the pupil-size deviation from baseline, in arbitrary units, for varying target eccentricities (of 5°, 10°, and 15°), separately for grouped (**left**) and ungrouped (**right**) target configurations. The dashed vertical lines denote the sequence of display frames on a given trial (fixation, premask, blank, search display, and dilation period, respectively). (**B**) Mean pupil size deviations from baseline (with corresponding within-subject 95% confidence intervals) for grouped (**left**) and ungrouped (**right**) targets as a function of target eccentricity, as measured in the dilation period (the gray shaded area in the figures in panel A). Note that the subtraction procedure used to calculate mean pupil-size deviations yielded negative values, where a larger negative deviation corresponds to a smaller pupil size (thus reflecting a comparably narrow attentional focus)

**Fig. 4:**
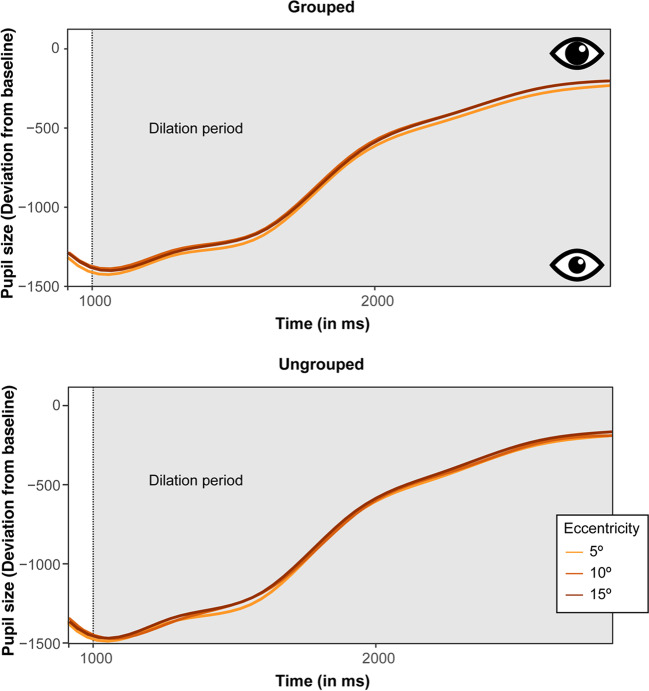
Time courses of the pupil-size deviation from baseline (in arbitrary units) during the dilation period at varying target eccentricities (of 5°, 10°, and 15°), for grouped (**top**) and ungrouped (**bottom**) target configurations

Figure 3A depicts the time courses of the pupil size deviations (relative to the baseline) for each target eccentricity, separately for the grouped (left) and ungrouped (right) target configurations (in arbitrary units). Moreover, Fig. 4 additionally plots the same time courses specifically during the dilation period. As mentioned above, only trials with correct behavioral responses were included in the analyses of the pupillary responses, in order to ensure that potential variations in the pupil size reflect actual target processing (and are not simply related to some error-related processes, see, e.g., Maier et al., 2019). Note that due to the subtraction procedure (see *Methods, Pupillometry*), all mean pupil deviations took on a negative value, with more negative values denoting smaller pupil sizes (and, accordingly, a narrower focus of attention).

To start with, it is instructive to take a look at the overall curves depicted in Fig. 3A: following the appearance of the fixation cross, the pupil at first slightly constricts relative to the baseline level before dilating in response to the black background. Next, upon the (250-ms) presentation of the premask display, the pupil constricts again due to the sudden onset of the six bright (i.e., white) placeholders. Note that we included the premask display to allow for a global orientation process as to where potential target (and distractor) items will subsequently appear. The pupil keeps constricting during the (200-ms) intervening blank period and over the (300-ms) exposure of the search array, responding to the bright target and distractor stimuli. And then, after the offset of the search array, the pupil dilates over the 1,750-ms “dilation” period during which only the fixation cross remains in view on a black screen background (see also Fig. 4). Thus, the pupil response is strongly light-driven during the first part of the trial, swamping the expression of any subtle covert attentional orienting processes (see also Brocher et al., 2018, for a similar modulation given a comparable trial sequence). Such processes only become observable in differences of the pupil size during the dilation period, with the fading of light response. This is not to say that the covert attentional processes that may be tracked by changes in pupil size commence only in the dilation period; rather, these processes are already set in motion during the presentation of the search array, but they would be “unmasked” only by the fading of the light response. Figure 3B presents the corresponding mean pupil-size deviations observed during the dilation period, for each condition.

Individual mean pupil-size deviations from baseline were again analyzed by means of a repeated-measures ANOVA with the factors Target Configuration and Eccentricity. While there was no main effect of Target Configuration, *F*(1, 28) = 0.92, *p* = .347, η^2^_G_ = .001, the main effect of Eccentricity was significant, *F*(1.66, 46.48) = 4.54, *p* = .021, η^2^_G_ = .01, with pupil size being overall smaller (indicative of a narrower focus of attention) when targets were presented closer to fixation (-202.58, -187.17, and -186.87 for eccentricities of 5º, 10º, and 15º, respectively). Importantly, the Target Configuration × Eccentricity interaction was also significant, *F*(2, 56) = 3.65, *p* = .032, η^2^_G_ = .01. Holm post hoc tests showed that for the grouped-target condition, there was a significant difference of the 5° eccentricity (*M* = -217.14) relative to both the 10° (*M* = -184.09), *p* < .001, and the 15° eccentricity (*M* = -191.05), *p* < .019. Thus, for grouped targets, the pupil size was significantly smaller when the target appeared close to fixation. For the ungrouped-target condition, eccentricity variations did not influence the size of the pupil, and the pupil diameter was overall comparable to the most distant, 15° eccentricity position in the grouped-target condition, *p*s > .05. Thus, the pupillometry data revealed a comparable pattern to the response accuracies, with a smaller pupil size (indicative of a narrower attentional focus) for grouped targets presented closer to fixation. For the ungrouped targets, the pupils were more dilated irrespective of target eccentricity (indicative of a rather broad focus of attention).

To further assess the specific dynamics of the pupillary response to grouped targets, we performed an additional analysis of the pupillometry data by subdividing the total (1,750-ms) dilation period into two halves: an early and a late dilation period (of 875 ms each). Separate analyses of both halves showed that the above-described constriction of the pupil for the grouped target at the near-fixation location emerged only late, in the second half of the dilation period (*ps* < .05 for the comparison of the 5° eccentricity with the 10° and 15° eccentricities), while not yet manifesting during the early period (all *ps* > .05 across all three eccentricities) – consistent with the “unmasking” notion outlined above. This pattern may thus be taken to indicate that attention was initially distributed rather broadly (to orient in the entire search array) before focusing upon the grouped target (at least when presented at a central location), thereby improving the resolution of attention for target-related processing.

Moreover, to additionally investigate whether the light-driven pupil response at the beginning of the dilation period might have influenced our pattern of results, an additional control analysis was performed (following a procedure previously employed by Wang et al., 2015). This analysis used the first 875 ms of the dilation period (i.e., the first half) as the baseline (instead of the 250-ms premask display) in order to quantify differences in the remaining time period when the effect of the light response has presumably faded. This analysis again revealed significant main effects of Target Configuration, *F*(1, 28) = 5.98, *p* < .05, η^2^_G_ = .004, and of Eccentricity, *F*(1, 28) = 5.27, *p* < .001, η^2^_G_ = .001, but this time no reliable interaction, *F*(2, 56) = 0.39, *p* = .679, η^2^_G_ < .001. Pupil sizes were smaller, thus revealing a narrower attentional focus with grouped (509.86) than ungrouped (541.59) targets, and they were also smaller when the target was presented closer to fixation than further away (514.91, 527.21 and 535.05 for eccentricities of 5º, 10º, and 15º, respectively). This result thus essentially replicates the above-described findings and again shows that the variations in the pupillary response indeed stabilized towards the end of the dilation period when the pupillary light response is less pronounced. Overall, this might be taken to indicate that our results are actually rather robust despite some changes in luminance during the trial sequence.

## Discussion

Employing a visual search task, Experiment 1 examined for variations of the attentional breadth as associated with the localization of more versus less grouped target configurations at varying eccentricities. The results revealed a grouping benefit that scaled with eccentricity: grouped targets appearing closer to fixation were detected with higher accuracy than more distant targets. No comparable benefit was found for ungrouped targets, which exhibited a level of performance overall comparable to the grouped target at the greatest eccentricity. The pupillometric data essentially mirrored this pattern; in particular, pupil sizes were smaller for grouped targets appearing closer to fixation, as compared to more dilated pupils for more distant grouped targets and for all ungrouped targets (irrespective of their eccentricity). Moreover, this constriction of the pupil for grouped targets close to fixation appeared to occur relatively late in time, in the second half of the dilation period. Together, this pattern of results shows that the observable grouping benefit covaries with the availability of attentional resources: Grouped targets at central locations elicit (after some time) a relatively narrow focus of attention and are detected with high accuracy, whereas more distant grouped targets (and ungrouped targets at all locations) require attention to be distributed more broadly across the entire trial while still being detected only with relatively low (though, with above-chance level) accuracy. Attention (as measured in the pupillometric data) thus appears to scale with the concurrent grouping demands. The attentional focus seems to be initially set broadly by default, covering a large area of the visual field, yet only at a relatively low resolution. After a broad scan of the array, the grouped target particularly triggers a narrowing of the focus, increasing the attentional resolution (and, correspondingly, performance; see Shepherd & Müller, 1989). In this view, grouping benefits performance in particular when a sufficient amount of attentional resources is available at the locations of the to-be-grouped items (see, Nowack et al., 2021). Of note, these grouping-dependent variations of attentional resolution also covaried with concurrent task effort, as already suggested above in the *Introduction*.

Interestingly, this result pattern would appear to be inconsistent with an alternative theoretical view, which assumes that attention is allocated upon the completion of preattentive-automatic grouping operations (e.g., Mattingley et al., 1997). That is, the preattentive integration of separate parts into a grouped object would enhance the saliency of that object (e.g., Kimchi et al., 2016; Rauschenberger & Yantis, 2001), as a result of which attentional resources would be attracted more strongly by the grouped, salient configuration. Such a process of essentially object-based attentional capture would be expected to be associated with focused attention being allocated towards the grouped, salient object early on during processing. However, in our experiment, attention was initially set broadly across the entire search array and focused only after some considerable delay. This pattern appears less consistent with the notion of an automatic (i.e., purely preattentive) attraction of attention by salient object groupings.

Experiment 2 was designed to further test the strength of the linkage between grouping and the availability of attention, that is, whether effective grouping depends on the amount of attentional resources available at the target location. As described above, grouped targets at near-foveal locations were detected very accurately with a narrow focus of attention, while performance dropped for more peripheral, grouped targets for which attention was more broadly distributed. This pattern might be taken to indicate that a certain amount of attentional resources has to be available in order to trigger effective object completion. This idea was further tested in Experiment 2 by combining peripheral search for a grouped/ungrouped target configuration with an attentionally demanding foveal task (see, e.g., Mack et al., 1992, and Moore & Egeth, 1997, for a similar logic). The addition of such a second task allowed us to assess peripheral search performance when attentional resources were partly unavailable (due to being occupied in the center), thereby impacting the allocation of attention to the lateral target grouping.

## Experiment 2

Experiment 2 was in most respects comparable to Experiment 1, except that a dual-task paradigm was implemented in order to reduce the amount of attentional resources available to process the peripheral target configurations. A given trial would again consist of an initial premask, followed (after some delay) by a search display (similar to Experiment 1). In addition, Experiment 2 consisted of two experimental parts, which were presented in counterbalanced order across participants: In the “*single-task*” part of the experiment, observers were required to discern the presence versus absence of a (grouped or ungrouped) lateral target among distractors. We deliberately introduced a (target present vs. absent) detection task in Experiment 2 (as compared to the left/right target-localization task used in Experiment 1) in order to rule out a potential strategy of restricting the search to only one half of the display. In more detail, in the localization task (as used in Experiment 1), monitoring the stimuli in only one display half would potentially allow observers to infer the left/right location of the target in the whole display, that is: if it can be ruled out that the target is not present on the searched side, it would have to be located on the opposite side (allowing a default “opposite-side” response). Such a possible strategy was avoided by introducing a target-detection task (in Experiment 2): the introduction of target-absent trials requires observers to search both display halves in order to accurately determine the presence (vs. the absence) of a target.

In the “*dual-task*” part of the experiment, the same peripheral target-detection task was used but it was additionally accompanied by a second, attentionally demanding foveal line-length discrimination task. To elaborate, together with the onset of the search display, the central fixation cross was presented with the crossing line segments stretched either vertically or horizontally, and observers were asked to report the orientation of this stretched cross (see Fig. 1E and Mack et al., 1992, for a comparable procedure). If detection of the grouped target would still reveal a benefit (relative to the ungrouped target), despite a substantial amount of attentional resources being occupied by the foveal task, this could be taken to indicate that (Kanizsa-type) grouping of the target fragments occurs even when only limited attentional resources are available to trigger object completion.

### Materials and method

Experiment 2 was by a large extent comparable to Experiment 1, apart from the following changes: The experiment was separated into two distinct parts. In the single-task part of the experiment, the sequence of events on a given trial was essentially comparable to Experiment 1 (see Fig. 1D), except that observers were now asked to report the presence versus absence of the target (rather than to left/right localize the target, as in Experiment 1). Observers were instructed to respond as accurately as possible, without time restrictions, by pressing the left [right] arrow key on the keyboard to target presence [absence], respectively. As mentioned above, the change from a localization to a detection task was implemented in order to prevent observers from simply using a strategy that bases the response on the monitoring of only one half of the display. In target-absent displays (one-third of all trials), six randomly oriented distractors would be presented. In target-present displays, the target would be located at the intermediate (10°-eccentricity) location in either the left or the right display half (with equal probability). Only one target eccentricity was used to ensure a sufficient amount of trials per condition, while maintaining an appropriate length of the experiment and to control for potential influences from crowding effects at variable target eccentricities. Moreover, presenting the target only at two, rather than six, possible locations should also make the task somewhat easier (observers were informed about the placement of the target before the experiment). Recall that in Experiment 1, the middle 10°-eccentricity location also exhibited a robust grouping benefit, justifying the use of this target eccentricity in Experiment 2. The remaining five other locations in target-present displays (and all six locations in target-absent displays) were again occupied by a distractor configuration (with randomly selected upward or downward orientation of the indents).

In the dual-task part of the experiment, the lateral search task was identical to the procedure in the single task. Critically, however, in an additional foveal task, the initially presented fixation cross (0.4° × 0.4°) changed the length of its arms during the presentation of the search display, revealing either a horizontally stretched cross, 0.5° × 0.3°, or a vertically stretched cross, 0.3° × 0.5° (see Fig. 1E). It should be noted that changing the cross dimension from 0.4° × 0.4° (in the single-task part) to either 0.5°× 0.3° or 0.3°× 0.5° (in the dual-task part) did not introduce an overall luminance change in the display center since the overall physical stimulation remained constant. Participants were asked to indicate whether the horizontal or the vertical line of the fixation cross was longer by pressing the left or right arrow keys, respectively. The response to this foveal task was issued by a response cue that presented the words “Which line was longer?” on the screen center, and which was presented on the screen *after* observers responded to the presence/absence of a lateral search target (which was issued following the response cue that presented the words “Was a target present?” on the screen center). Observers were thus provided with an identical trial sequence in the lateral search task in both the single- and the dual-task conditions. They were instructed to prioritize this new, foveal judgment task over the lateral search task.

A new sample of 30 participants (11 males; mean age 26.77 (*SD =* 6.83) years) with normal or corrected-to-normal vision took part in Experiment 2, either for course credits or payment (9 €). The sample size was again determined on the basis of the above-described power analysis. For the eye-movement recordings, the sampling frequency was reduced to 250 Hz (see Brocher et al., 2018) to prevent high levels of noise during data acquisition. Central eye gaze was once again monitored, and a given trial was discarded when a saccade (indicative of an overt orienting response) was made (2.6% of all trials).

The order of the single- and dual-task parts of the experiment was counterbalanced across participants where each part consisted of four blocks, with short breaks in between. Each block presented 60 trials in randomized order: 20 target-present/grouped, 20 target-present/ungrouped, and 20 target-absent trials. On target-present trials, the target was equally likely to appear in the left or right display half. In the dual-task part of the experiment, the horizontally or vertically oriented fixation cross appeared with equal probability. Experiment 2 consisted of 480 trials overall, with two experimental factors: target configuration (grouped, ungrouped, absent) and task load (single, dual task). The total experiment lasted approximately one hour, including the instruction of the participants, a short practice session (of at least 18 trials) at the beginning of each experimental part, and the eye-tracker calibration routine at the beginning of each block.

Preprocessing of the pupillometry data followed the same routines as in Experiment 1, which led to the exclusion of 6.1% of all trials.

## Results

### Response accuracy

To ensure that attention was engaged in the foveal line-discrimination task, we only analyzed trials in which the orientation of the central fixation cross was correctly identified (92.4% of all trials). Moreover, target-absent trials (which yielded overall 78.1% correct responses, with more accurate responses under the single- as compared to the dual-task load, 84.8% vs. 71.4%, respectively, *t*(29) = -4.95, *p* < .001) were also excluded from the data proper before the analysis of the lateral target detection accuracies.

**Fig. 5:**
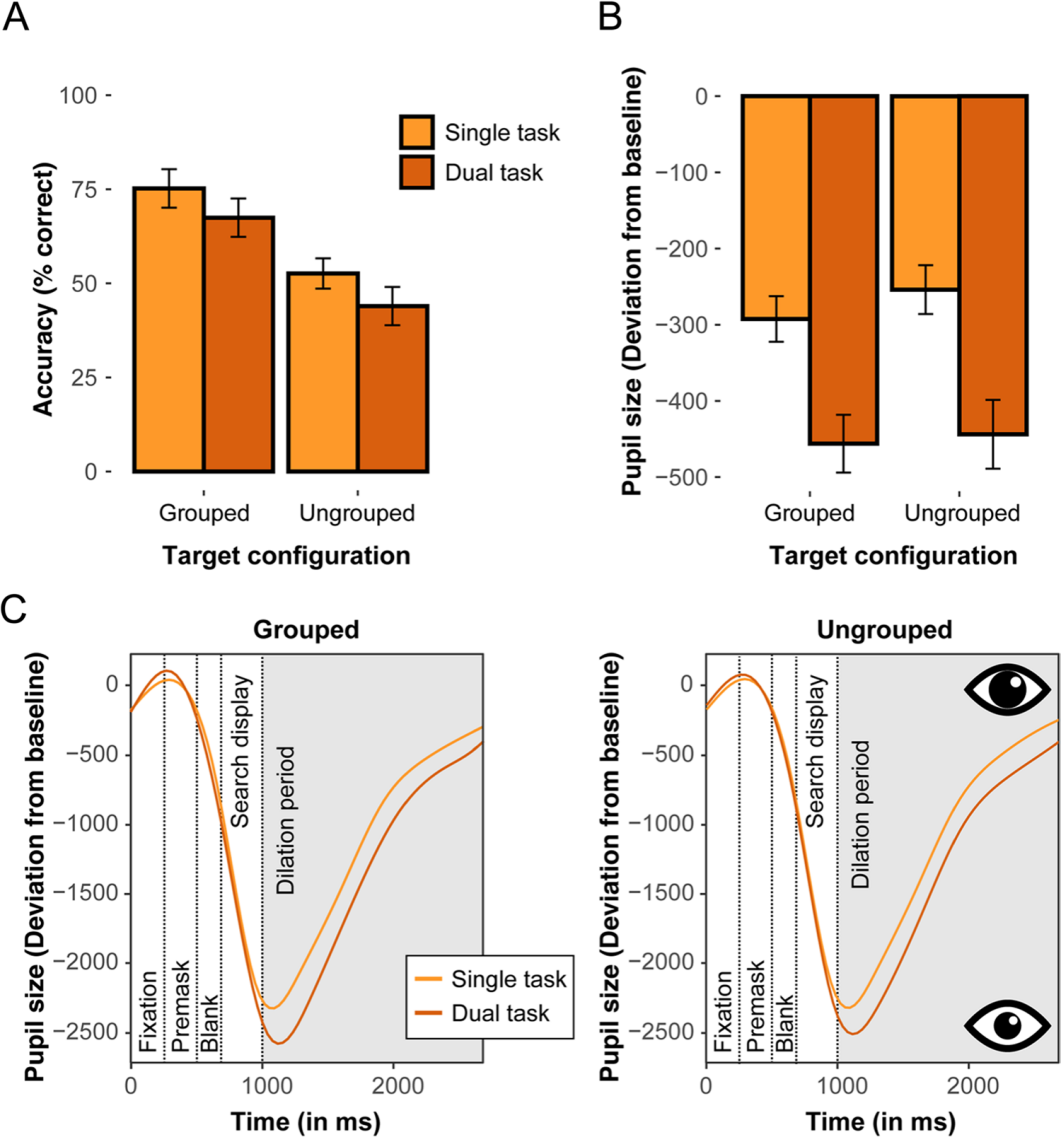
Results in the peripheral search task in Experiment 2 (given correct performance in the foveal task under dual-task conditions). (**A**) Mean accuracies (% correct) and (**B**) mean pupil size measures (with corresponding 95% within-subject confidence intervals) for grouped and ungrouped targets as a function of task. Pupil size measures depict the deviations from baseline as measured in the dilation period. (**C**) Time courses of the pupil-size deviation from baseline, in arbitrary units, in the single- and dual task conditions for grouped (left) and ungrouped (right) target configurations. The dashed vertical lines denote the sequence of display frames on a given trial (fixation, premask, blank, search display, and dilation period, respectively). Note that the use of a subtraction procedure to calculate mean pupil deviations resulted in negative values, with a larger negative deviation corresponding to a smaller pupil size (thus reflecting a comparably narrow focus of attention)

Figure 5A presents the mean accuracies in the peripheral search task for the two types of target configuration as a function of task load. Individual mean accuracies were analyzed using a repeated-measures ANOVA with the factors Target Configuration (ungrouped, grouped) and Task Load (single task, dual task). This analysis revealed a main effect of Target Configuration, *F*(1, 29) = 40.32, *p* < .001, η^2^_G_ = .28, with overall higher response accuracies (by 23.4%) for grouped than for ungrouped targets (which is essentially comparable to performance for the middle 10º position in Experiment 1, where the grouped target revealed a comparable benefit of 21.2% relative to the ungrouped target,* t*(29) = 0.62, *p* > .05). The main effect of Task Load was also significant, *F*(1, 29) = 7.87, *p* = .008, η^2^_G_ = .04: responses were more accurate overall under single- (63.9%) than under dual-task (55.5%) conditions; that is, having to perform the foveal task indeed resulted in a substantial reduction of performance on the peripheral search task. The target configuration by task load interaction was not significant, *F*(1, 29) = 0.30, *p* = .586, η^2^_G_ = .006. Thus, the grouped target substantially improved performance (relative to the ungrouped target) both when attention was fully available (in the single-task condition) and when a rather large amount of attention was absorbed by the secondary, foveal task (in the-dual-task condition).

As in Experiment 1, we again arcsine-transformed the accuracy data to improve normality and to exclude the possibility that the nonsignificant interaction in the ANOVA was caused by a floor effect. This analysis revealed significant main effects of Target Configuration, *F*(1, 29) = 42.07, *p* < .001, η^2^_G_ = .29, and Task Load, *F*(1, 29) = 7.58, *p* = .010, η^2^_G_ = .05 but again no significant interaction (p > .05), thus mirroring the above-described results.

### Pupillometry

**Fig. 6:**
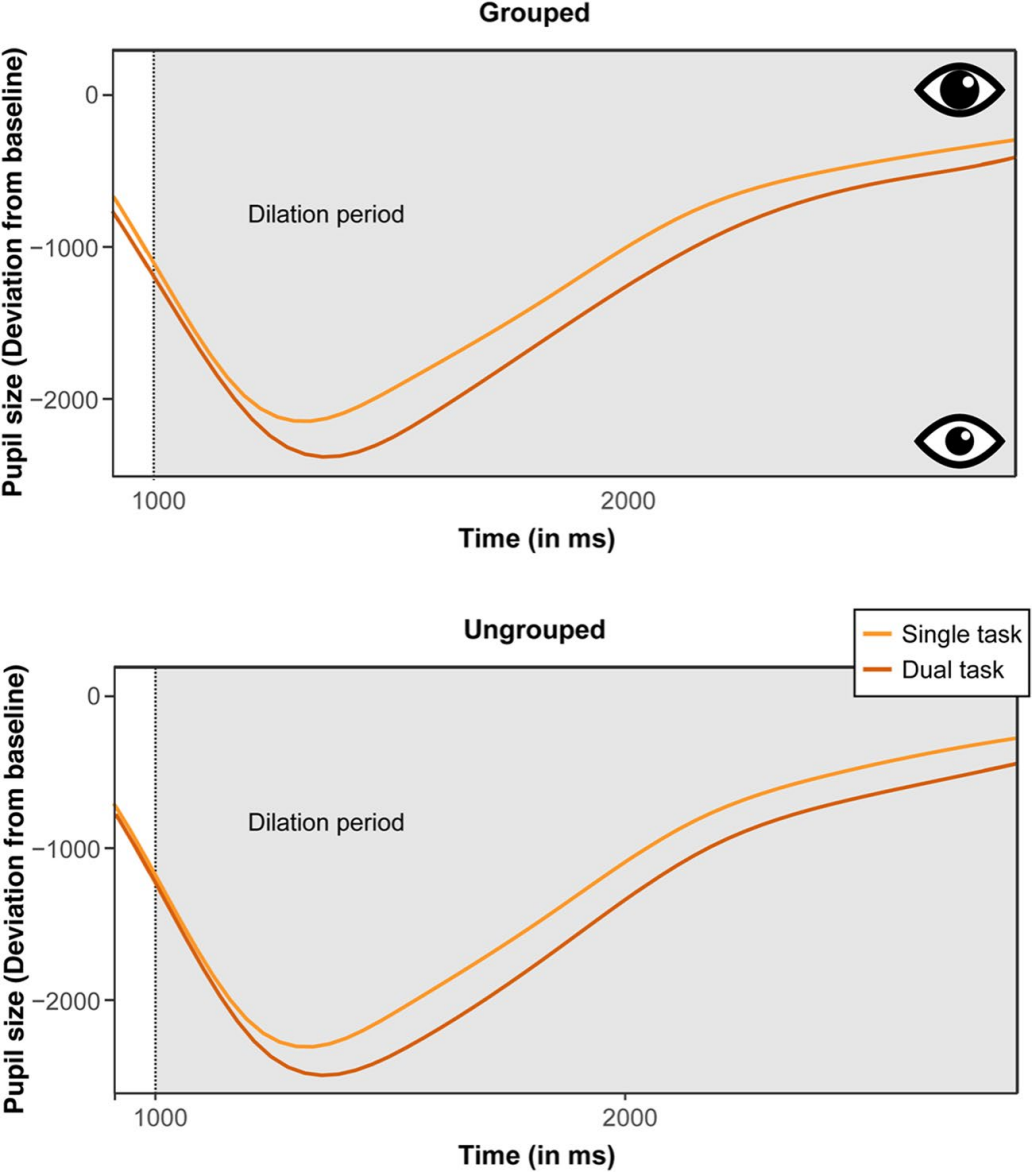
Time courses of the pupil-size deviation from baseline (in arbitrary units) during the dilation period, in the single- and dual task conditions for grouped (**top**) and ungrouped (**bottom**) target configurations

Figure 5B presents the mean pupil-size deviations from baseline, for each experimental condition during the dilation period (analogous to the procedure described in Experiment 1). Moreover, Fig. 5C depicts the time courses of the pupil size deviations (relative to the baseline) for each task load, separately for the grouped (left) and ungrouped (right) target configurations. Figure 6 additionally plots the same time courses only for the dilation period. Recall that due to the subtraction procedure, all mean pupil deviations revealed negative values, where more negative values indicate that the pupil size became smaller, indicative of a narrower focus of attention. Trials with incorrect behavioral responses (in both the central discrimination and the peripheral detection task) were again discarded from the data proper (to ensure that pupil size variations reflect processing of the target and are not contaminated by error-related variations). Individual mean pupil-size deviations were analyzed by a repeated-measures ANOVA with the factors Target Configuration and Task Load.^2^ The results revealed a main effect of target configuration, *F*(1, 29) = 9.42, *p* = .004, η^2^_G_ = .01, with smaller pupil sizes for grouped (*M* = -374.26) versus ungrouped targets (*M* = -348.84). There was also a significant main effect of task load, *F*(1, 29) = 31.75, *p* < .001, η^2^_G_ = .13 with the pupil size being markedly smaller when participants had to focus on a second, foveal task (*M* = -449.95), as compared to the single-task condition (M = -273.15). Importantly, however, the Target configuration × Task Load interaction was not significant, *F*(1, 29) = 1.42, *p* = .243, η^2^_G_ < .001. Overall, the pupillometry data thus revealed a comparable pattern of results as for the response accuracies, showing a clear effect of task load: Attention was focused more strongly at central locations when the additional foveal task had to be performed (evidencing the resource-demanding nature of the central task). However, there was also a grouping benefit: attention was more focused when a grouped target was presented than when the target was ungrouped. This grouping benefit in the pupillometry data was essentially independent of the task load.

## Discussion

In Experiment 2, a secondary foveal task was introduced to investigate how the availability of attentional resources would impact processing of the lateral search items. The results showed that the foveal task was indeed successful in binding attentional resources, rendering search for the lateral targets less accurate. Nevertheless, detection of the grouped target exhibited a reliable performance benefit relative to the ungrouped target and this grouping benefit was independent of whether or not a foveal task had to be completed.

Concurrent measures of pupil size again were comparable to behavioral performance, with more dilated pupils for the single- as compared to the dual-task condition. This indicates that the amount of available resources and their potential allocation to the peripheral search items was directly reflected in the changes of the pupil diameter, with an overall stronger central attentional focus under dual-task conditions (despite a more difficult task). Moreover, there was an effect of grouping on the pupillometric data: pupils were more dilated for ungrouped than for grouped targets (comparable to Experiment 1), independently of the task load. This indicates that search for ungrouped targets is associated with a broader distribution of attentional resources (that comes along with a lower attentional resolution) than search for grouped targets, likely because grouped targets summon the available attentional resources more efficiently than the corresponding ungrouped targets. Importantly, this benefit for the grouped target was already evident when only a limited amount of attentional resources was available to trigger object completion. Thus, as in Experiment 1, in Experiment 2, grouping-dependent pupillary variations scaled with the breadth of attention and its concurrent changes in task effort. However, the comparison across single- and dual-task loads additionally showed that smaller pupil sizes in the dual task reflected a narrower attentional focus (than in the single task), even though the dual task was more demanding. Since more demanding tasks would usually be associated with an increased pupil size (e.g., Beatty, 1982), this latter finding thus indicates that the resolution of attention may be dissociated from the difficulty of a given task under specific circumstances.

While a benefit of grouping appeared to occur independently of whether attention was partly focused in the display center or not, the overall performance accuracy in the single-task condition of Experiment 2 (63.9%) was somewhat lower than performance for the same 10°-eccentricity target position in Experiment 1, which yielded a mean accuracy of 73.8%, *t*(29) = 5.39, *p* < .001. This enhanced performance in Experiment 1 may be due to the somewhat easier localization task, and the blocked presentation of the target configurations, which presumably helped observers prepare for an upcoming trial. However, even though there were some minor differences in task difficulty, it should be noted that the overall grouping benefit (of around 20%) was comparable across both experiments.

## General discussion

The current study investigated whether perceptual grouping can facilitate visual search and whether such a grouping benefit would vary with the amount of available attentional resources. Our experiments were in part motivated by recent findings from experiments with neuropsychological patients who showed deficits of selective attention due to parietal brain damage, and which revealed that object grouping was ineffective in parts of the visual field where attention was lacking (Nowack et al., 2021; see also Conci et al., 2018; Gögler et al., 2016), the theoretical implication being that effective perceptual grouping depends on the availability of attentional resources. To validate these previous findings and extend them to healthy observers, the current study tracked the engagement of covert attention by measuring pupil dilations while systematically comparing visual search for grouped versus ungrouped targets with targets appearing either at varying eccentricities relative to central eye-fixation (Experiment 1), or during a concurrent, attention-demanding central (foveal) discrimination task (Experiment 2).

Experiment 1 used a visual search task that required participants to localize a grouped or, respectively, ungrouped Kanizsa-type target among distractor configurations in peripheral vision. The behavioral results revealed grouping to facilitate target localization: response accuracies were higher for grouped (79.2%) than for ungrouped target configurations (62.2%). This essentially replicates previous findings showing that grouping by collinearity and closure may lead to an increase of the conspicuity of the Kanizsa target figure, thereby facilitating search (Conci et al., 2007; Conci et al., 2011; Kimchi et al., 2016; Nie et al., 2016; ; Pomerantz & Portillo, 2011; Wiegand et al., 2015). Moreover, the performance benefit for grouped targets was dependent on the eccentricity at which the target was presented: at an eccentricity of 5°, grouped targets were localized with very high accuracy (91%), but accuracy dropped monotonically with increasing distance from fixation (to 84% and 62% at eccentricities of 10° and 15°, respectively). It is typically assumed that the availability of attention is highest in central vision and decreases with increasing distance from the fovea (Ducrot & Grainger, 2007; Jacobs, 1979). Moreover, when attention is distributed over a larger area of the visual field, its resolution decreases, as compared to when attention is more narrowly focused (Eriksen & Yeh, 1985). The eccentricity effect in the current experiment may thus indicate that the efficiency of grouping scales with the gradient of attentional resolution. By contrast, performance for the ungrouped targets was relatively low throughout (e.g., only 65.2% at the most central, 5° position), indicating that an object that is not grouped is also not processed more efficiently when more attentional resources are available (i.e., closer to fixation). Together, this pattern of results lends support to the idea that successful grouping (as evidenced in variations of our measure of behavioral performance) is linked to the availability of attentional resources, which are particularly concentrated in more central vision and which scale with changes of the attentional breadth.

This conclusion is also supported by our pupil-size data. Previous studies showed that the pupillary light reflex reveals a constriction of the pupil in response to brightness and a concurrent dilation in response to darkness (Mathôt, 2018). Moreover, it was reported that covertly attending to a bright or dark stimulus elicits a comparable pupillary light reflex as if one would be looking directly at a given stimulus, albeit with a much weaker modulation (Binda & Murray, 2015; Binda et al., 2013, 2014; Mathôt et al., 2013, 2014). Covert changes of the attentional breadth were also evident in the current study, as participants had to attend to bright stimuli in the periphery without making any eye movements (however, these changes in allocating attention occurred in the absence of luminance manipulations, since all stimuli always had the same amount of light entering the eyes, i.e., there was no bright vs. dark stimulus manipulation). In the dilation period, after the presentation of the stimuli, we observed systematic differences in pupil size, depending on the type of target and eccentricity. For instance, with grouped targets, the pupil was dilated initially, while it constricted towards the end of the dilation period for the proximal target at 5°, suggesting that attention is initially distributed broadly in order to attend to and process the more distant stimuli, while the central, grouped target is then focused later on. For correctly localized targets in the ungrouped target condition, however, the pupil diameter was overall largely constant and comparable to the most peripheral, 15° position in the grouped target condition. Overall, these results (in particular with grouped targets) accord with the findings of Brocher et al. (2018), who reported that the size of the pupil varies in relation to stimulus-to-fixation distance when participants covertly shift attention to peripherally presented stimuli. Similarly, it has also been shown that the pupil is also more dilated when attention spreads more broadly as compared to when a more narrow focus of attention is required (Daniels et al., 2012; Ivanov et al., 2019; for a review, see Mathôt, 2020).

Together, the pupil-size effects in Experiment 1 thus show that task effort and its associated variations of the attentional focus scales with the benefit of grouping in the target: attention appears to be initially distributed broadly by default, covering a large area of the field, though only at a relatively low resolution. After a broad scan of the array, the grouped target triggers a narrowing of the focus (evident especially with a target at 5º eccentricity), increasing the attentional resolution. By contrast, ungrouped targets are processed comparably inefficient at all eccentricities.

Alternatively, one could argue that the efficiency of grouping scales with visual acuity. In central (foveal) vision, the concentration of cones is very high and then decreases at eccentricities beyond 10º of visual angle (Pouget, 2019). Thus, high visual acuity is only available in foveal and peri-foveal vision, which, however, already encompassed the two inner-most target locations in our search displays. From a visual-acuity perspective, stimuli at larger eccentricities should elicit poorer performance than more proximal ones independently of their configuration. In Experiment 1, however, we found no significant difference in performance for ungrouped target configurations across all three eccentricities (there was an eccentricity-dependent effect only for the grouped targets), which renders it unlikely that the results are due to an overall gradient of visual acuity. Moreover, Brocher et al. (2018) also assessed whether the cortical magnification factor and associated variations of visual acuity (across a rather large range of eccentricities of up to 42.5º) may lead to variations in pupil size (independently of concurrent attention shifts). Their results, however, indicated that differences in eccentricity and related changes in the density of photoreceptors in the retina cannot explain the observed change in performance; instead, the observed variations in accuracy as a function of eccentricity can be related primarily to concurrent attention shifts.

In Experiment 2, the availability of attentional resources in the periphery was further restricted by means of an attention-demanding foveal discrimination task. The question was whether the grouping benefit in the peripheral search task would still be evident when a substantial amount of attentional resources is bound elsewhere. The behavioral results again showed that grouping facilitates target detection: response accuracies were by 23.4% higher for grouped than for ungrouped target configurations. Moreover, performance was affected by the attention-binding foveal task: response accuracies were reduced, by 8.4%, under the dual- relative to the single-task load. Importantly, however, the grouping benefit manifested (and its magnitude was independent of) whether or not observers had to perform the attention-binding foveal task. This shows that grouping in the peripheral target can still give rise to a benefit even when only rather limited attentional resources are available. The pupil-size data again mirrored the behavioral results: the pupil size was overall smaller when participants performed the search under dual-task conditions, as compared to the single task where more attentional resources were available to process the targets in the periphery. This finding is overall consistent with findings showing that a high attentional load at the fovea goes along with a comparably narrow attentional focus (Daniels et al., 2012; Kornrumpf & Sommer, 2015). Moreover, as in Experiment 1, detection of grouped targets was associated with smaller pupil sizes compared to ungrouped targets, again indicating that grouped targets are not only easier to detect but also summon attention more efficiently than ungrouped target stimuli. The results from Experiment 2 thus show that a grouping can facilitate performance and modulate covert attention spreading even when only residual attentional resources are available.

Previous studies that employed a foveal task to bind attentional resources have revealed that observers are unable to identify a grouping pattern in the periphery when they were not attending to these groupings (Mack et al., 1992; but see Moore & Egeth, 1997). This may be taken to suggest that object-based selection operates only within spatially attended regions (Lavie & Driver, 1996). However, extended practice (Ben-Av et al., 1992; Li et al., 2002) and the expectation to report such a grouping pattern (Chan & Chua, 2003; Mack et al., 1992) would typically make observers adopt a strategy of dividing their attentional resources so as to adequately process both stimuli in the foveal and the concurrent peripheral task. While such a division of attention may only leave a limited amount of resources available to process the peripheral stimuli, such processing of information in the “near absence of attention” has nevertheless been shown to reveal well above-chance performance in a relatively complex object-categorization task (Li et al., 2002). Overall, these previous findings are thus compatible with the current results. Our participants were explicitly told to perform a dual task, that is, to classify the central stimulus *and* search for a target in the periphery. Accordingly, one would expect that they saved at least some attentional resources for the search task, and such processing of the search items in the near-absence of attention (i.e., given only residual attentional resources) apparently sufficed to generate a reliable grouping benefit. Essentially, this grouping benefit arising on the basis of only residual attentional resources may be comparable to the effects seen in neuropsychological patients, where effective grouping was likewise found to depend on the availability of at least some attentional resources in the otherwise neglected visual field (Conci et al., 2018; Gögler et al., 2016; Nowack et al., 2021).

Across both experiments, search for the ungrouped targets revealed overall a relatively low level of performance and a comparably broad tuning of the attentional focus (as reflected in the pupil-size measures). There were also no eccentricity-dependent changes in both accuracy and pupil-size measures. A potential explanation for this absence of a modulatory effect might be that detection of this type of configuration was not facilitated by grouping processes, so that search had to be based on processing the arrangement of the individual inducer elements (Conci et al., 2007). That is, this task required a high amount of attentional resources to be performed successfully. Grouped targets, by contrast, provided a regular and simple structure requiring a much lower amount of attentional resources in order to trigger the grouping process and summon attention.

Taken together, our results thus show that part-to-whole object integration and search guidance by salient, integrated objects scale with the amount of available attentional resources. Our study also demonstrates and provides additional evidence that measurement of pupil size provides a useful method for investigating changes of the distribution of attention beyond basic variations of physical stimulus intensity.

## Footnotes

1 Note that an important difference to the study of Mack et al. (1992) is that the peripheral stimuli in our experiment were task relevant. It could therefore be assumed that a certain (possibly, a rather small) amount of the available attentional resources was still devoted to the peripheral stimuli despite the central task load. By contrast, Mack et al. (1992) only tested processing of an irrelevant stimulus on a single “surprise” trial, thus effectively inducing “inattention.”

2 Note, that target-absent trials were also excluded from the data proper before the main analysis of the pupil sizes in target present trials. However, an additional analysis of target-absent trials also revealed a significant difference in pupil size between single- (*M* = -295.13) and dual-task (*M* = -433.39) conditions, *t*(29) = 5.69, *p* < .001.
